# Sirtuins at the Breaking Point: SIRT6 in DNA Repair

**DOI:** 10.18632/aging.100014

**Published:** 2009-01-20

**Authors:** David B. Lombard

**Affiliations:** Department of Pathology and Institute of Gerontology, University of Michigan, Ann Arbor, MI 48109, USA

Aging is the accumulation of unrepaired damage to cellular and organismal components over time. Damage to nuclear DNA likely contributes to the degenerative effects of aging; unlike other cellular constituents, nuclear DNA cannot be replaced [1]. A wide spectrum of DNA lesions and associated repair pathways exist [1]: non-helix distorting lesions, such as those induced by oxidative damage, are repaired via base excision repair (BER); whereas helix-distorting base changes, like those caused by UV, are fixed via nucleotide excision repair (NER) and its subpathways. DNA double strand breaks (DSBs) represent a particularly severe challenge to the cell; if left unrepaired these lesions can induce cell death, replicative senescence, or conversely promote oncogenic transformation. For this reason, cells have evolved multiple pathways to repair DSBs: classical non-homologous end-joining (C-NHEJ), homologous recombination (HR), and other pathways such as alternative NHEJ [2,3,4]. In this issue of *Aging*, workby Chua, McCord et al. suggests that SIRT6, a member of a protein family previously implicated in promoting longevity, may function at least in part via increasing efficacy of DNA repair.

In eukaryotic cells, DNA does not exist as a naked double-stranded molecule; rather it is packaged with histones and other proteins into a complex structure called chromatin [5]. Transcription, replication, and repair factors must navigate this environment to interact with DNA. Covalent modification of N-terminal histone tails (via phosphorylation, methylation, ubiquitination, sumoylation, ADP-ribosylation, and acetylation) leads to alterations in chromatin function. Chromatin itself may serve as a target of age-related change; significant alterations in chromatin structure occur during senescence of mammalian cells *in vitro*, and perhaps in the context of the whole organism as well [5]. One consequence of accumulated DNA damage and/or chromatin aberrations seems to be age-related perturbations in gene expression in some tissues [5,6,7], which may in turn lead to progressive loss of cellular and organismal homeostasis. However, these transcriptional changes are not found in all cell types [8], implying that different tissues vary with respect to their maintenance of an appropriate gene expression pattern, and potentially chromatin structure, with age.

In budding yeast and higher organisms, homologs of the Sir2 protein (the sirtuins) link chromatin structure with lifespan [9,10]. In yeast, transcription and recom-bination of certain regions of the genome - the telomeres, the ribosomal DNA array, and the silent mating type loci - are down-regulated through the action of Sir2 and multiple other factors. Sir2 possess histone deacetylase and ADP-ribosyltransferase activities, both dependent on the metabolic cofactor NAD^+^. In general, histone deacetylation promotes a more compact chromatin structure and inhibits transcription and recombination. In yeast, overexpression of Sir2 increases replicative lifespan - that is, the longevity of a single yeast mother cell - whereas deletion of Sir2 shortens it [11]. Sir2 possesses at least two functions in yeast relevant for longevity. First, Sir2 suppresses recombination at the repetitive array encoding the ribosomal rRNA (the rDNA locus) [5]. HR-mediated excision and subsequent replication of extra-chromosomal rDNA circles is an important cause of replicative aging in this organism. Second, Sir2 regulates the distribution of oxidatively damaged proteins [12]. In wild-type cells, oxidized proteins are specifically partitioned to the mother cell, so that each daughter cell emerges with a complement of undamaged proteins and suffers reduced levels of oxidative stress; in Sir2 mutants this process fails to occur.

A great deal of excitement has emerged from the observation that sirtuins in higher organisms are also involved in promoting longevity. In *C. elegans* and *D. melanogaster*, sirtuin overexpression or hyperactivity increases lifespan [13,14,15]. The mechanisms by which sirtuin action increases longevity appear to be species-specific; aberrant rDNA recombination has not thus far been implicated in limiting lifespan in any organism aside from budding yeast. However, in flies, Sir2 lies downstream of the histone deacetylase Rpd3 in lifespan extension [13], suggesting that, at least in this organism, chromatin represents the relevant Sir2 target in the context of longevity.

Mammals possess seven sirtuins, called SIRT1-SIRT7 [16]. Unlike yeast Sir2, which is only known to modify histones, mammalian sirtuins have evolved to target a plethora of distinct protein substrates, modulating a wide variety of biological processes: metabolism, cell survival, development, chromatin dynamics, DNA repair, and other phenomena [9,10]. Among sirtuin-deficient mouse strains, the SIRT6 knockout is particularly relevant in the context of the study of aging [17,18]. SIRT6 is found bound to nuclear chromatin [18]. In cells, deletion of SIRT6 results in genomic instability, manifested as chromosomal breaks and fusions, as well as sensitivity to specific genotoxins: the alkylating agent methyl methanesulfonate, hydrogen peroxide, and ionizing radiation, but not UV. This is a spectrum of sensitivities associated with defects in BER; indeed, these sensitivities can be rescued via introduction of a fragment of polymerase beta (polb), the major polymerase involved in "short-patch" BER. These observations suggest that SIRT6 might be involved in the BER process itself, either by modifying BER factors to promote repair or by modulating chromatin structure to permit access to DNA lesions. No interactions between SIRT6 and BER factors have been observed to date however; nor does SIRT6 have an apparent role in deacetylating polb itself *in vivo* [18]. In mice, the phenotype of SIRT6 deficiency is dominated by metabolic defects [18]. SIRT6 knockout mice are born at a Mendelian ratio and, though smaller than littermate controls, are fairly normal during the first two weeks of life. Subsequently, these mice begin to suffer from a complex metabolic/degenerative syndrome, with progressive severe hypoglycemia, lymphocytic apoptosis and wasting, culminating in death by four weeks of age.

How does the lack of SIRT6 produce such pleiotropic effects? It was originally shown that SIRT6 possesses ADP-ribosyltransferase activity [18,19]. More recently, Chua, Michishita and colleagues showed that SIRT6 has deacetylase activity as well, specifically targeting histone H3 on lysine 9 (H3K9) [20]. Knockdown of SIRT6 (S6KD) in human cells led to premature senescence coupled with genomic instability, although the pattern of instability - primarily involving telomeres - was distinct from the general instability observed in SIRT6-deficient mouse embryonic fibroblasts and ES cells [18]. The authors suggested that an altered chromatin state at telomeres in S6KD cells due to increased levels of acetylated H3K9 prevents association of factors required for proper telomere maintenance. One of these factors may be WRN, the protein defective in Werner syndrome, a disease with some manifestations resembling premature aging. Chua and colleagues found that WRN's association with telomeric DNA was greatly reduced in S6KD cells. This model is unlikely to explain the effects of SIRT6 deficiency in the mouse, however. The cellular and metabolic defects observed in the SIRT6 knockout mouse do not resemble those occurring with telomere maintenance defects, either in mouse or human, nor do they resemble the effects of WRN deficiency in either species [21]. The long mouse telomere reserve relative to human may explain some of these discrepancies.

In this issue of *Aging*, Chua, McCord and colleagues have now extended their characterization of SIRT6 function to a more general assessment of the role of this factor in DNA repair. They find that the association of SIRT6 with chromatin increases following induction of DNA damage, whereas overall levels of acetylated H3K9 decline in a SIRT6-dependent manner. In human cell lines, SIRT6 interacts with DNA-PKcs, a protein involved in NHEJ. Chromatin-associated DNA-PKcs also increases upon DNA damage in a SIRT6-dependent manner. In order to define more precisely the potential role of SIRT6 at DNA lesions, Chua and colleagues employ the meganucleases I-PpoI and I-SceI, which generate a few hundred DSBs (in the case of I-PpoI), or a single DSB following introduction of an appropriate site into the human genome (in the case of I-SceI). Using these systems, the authors find that DNA-PKcs is enriched around the site of a DSB; again this effect is dependent upon catalytically active SIRT6. Moreover, S6KD cells show defective DSB repair, as assessed by comet assay, although repair is normal when measured in SIRT6-immunodepleted extracts *in vitro*. The authors suggest that SIRT6 may function to deacetylate H3K9 at chromatin surrounding DSBs, potentially modulating access for DNA-PKcs and other repair factors.

This study is reminiscent of work in lower other organisms implicating sirtuins in DNA repair, as well as more recent studies on mammalian SIRT1 (see below). In yeast, loss of Sir2 leads to defective NHEJ indirectly, via silencing of essential end-joining factors such as Nej1 [22]. Loss of the sirtuins Hst3 and Hst4, which target acetylated H3K56, promotes genomic instability and DNA damage sensitivity [23,24,25,26]. In *T. brucei*, the sirtuin TbSIR2RP1 both deacetylates and ADP-ribosylates histones to promote DNA damage resistance, associated with increased bulk chromatin accessibility [27].

The study by Chua and colleagues raises a number of questions, particularly in light of previous characterization of the mouse SIRT6 knockout [18]. Mice deficient in DNA-PKcs or other NHEJ factors show profound defects in lymphocyte development as a consequence of a failure to rejoin RAG-mediated DNA breaks generated during immunoglobulin gene rearrangement [28]. However, SIRT6-deficient mice possess a normal lymphocyte complement prior to the onset of apoptosis [18]. Chua et al. suggest that lymphocyte-specific factors, such as the RAG proteins themselves, may compensate for the lack of SIRT6 during lymphocyte development. Other explanations are conceivable; for example, loss of SIRT6 may confer only a partial defect in DNA-PKcs function insufficient to impair gross lymphocyte development. One other discrepancy between this work and the previous study of the SIRT6 knockout concerns the nature of the DNA repair defect. While SIRT6-deficient mouse cells do not show impaired resolution of DSBs, human S6KD cells do demonstrate such a defect. The authors suggest that this discrepancy may reflect differing sensitivities of the assays used (pulsed field gel electrophoresis in the previous work versus comet assay in the current study). Equally well, these differences may reflect cell type-specific and/or species differences in SIRT6 function. To resolve these questions, it will be extremely helpful to assess SIRT6 and DNA-PKcs recruitment to DSBs, as well as DSB repair, in primary mouse cells using assays identical to the ones performed by Chua and coworkers.

More generally, in order to provide further mechanistic detail concerning the functional role of SIRT6 at chromatin in the context of DNA repair, it will be necessary to determine whether SIRT6 is recruited genome-wide in response to DNA damage, or locally, at the site of a DSB; as well as to assess the relationship between potential local SIRT6 recruitment and acetyl-H3K9 levels. The data presented do not provide insight into these issues. Moreover, in defining the role of SIRT6 in DSB repair, it would be helpful to perform studies to clarify the kinetics and order of SIRT6 recruitment relative to DNA-PKcs, as well as the kinetics of DSB repair in S6KD cells relative to controls (as opposed to testing at a single timepoint). Finally, given the data linking SIRT6 to BER function in mouse cells, it will be very informative to assess whether SIRT6 might promote association of other repair factors, such as those involved in BER, to DNA lesions, as the authors suggest.

Two recent studies on SIRT1 are relevant to the findings of Chua et al. Seto, Yuan and coworkers find that SIRT1 deacetylates and activates NBS1, an upstream DSB sensor [29]. More recently, Sinclair, Oberdoerffer and colleagues have described a role for SIRT1 in DNA repair and age-related alterations in gene expression [5]. They find that the association of SIRT1 with chromatin, like that of SIRT6, increases in response to DNA damage. Under basal conditions, SIRT1 is associated with a subset of promoters; induction of DNA damage leads to relocalization of SIRT1 protein and derepression of many of these SIRT1 target genes. SIRT1 is required for optimal HR and NHEJ, and, consistent with a direct role for SIRT1 at sites of DNA breaks, SIRT1 is recruited at these sites and is required for optimal recruitment of NBS1 and the HR factor RAD51. This function of SIRT1 may be important in aging; SIRT1 target promoters are derepressed during brain aging, a change blocked by overexpression of SIRT1. The authors suggest that over the lifetime of the cell, this relocalization of SIRT1 in response to DNA damage may lead to age-related perturbations in gene expression. Overall, SIRT1 appears to be involved in both modulating gene expression as well as directly recruiting factors to sites of DSBs.

These results regarding SIRT1 raise additional questions about the role of SIRT6 in DSB repair, and organismal homeostasis overall. What is the nature of the signal that triggers increased SIRT6 association with chromatin in response to DNA damage? ATM kinase and histone H2AX, two proteins involved in the DSB response, are required for SIRT1 recruitment in this context [5]. Is acetyl-H3K9 the only relevant SIRT6 target or, like SIRT1, does SIRT6 modify other proteins during this process? Could SIRT6 modulate transcription of genes relevant for DSB repair, either in the basal state or following genomic insult? In this context, SIRT6 emerged as a factor required for transcriptional repression in a screen for genes involved in silencing of the Fas promoter [30]. More recently, a study by Chua, Chang, Kawahara and colleagues demonstrated that SIRT6 plays a role in attenuating NF- κB signaling via H3K9 deacetylation [31]. Thus, SIRT6 clearly plays a role in modulating gene expression. Acetylated H3K9 is associated with NER [32], a repair pathway seemingly not involving SIRT6, and also marks actively transcribed promoters [33]. Thus, it is possible that the SIRT6-dependent bulk decrease in H3K9 acetylation in response to DSBs observed by Chua and colleagues reflects overall transcriptional alterations occurring in response to genomic insult, instead of or in addition to changes in histone acetylation specifically at sites of damage.

Perhaps the most fascinating question in this area concerns what the relationship is, if any, between the profound metabolic abnormalities of SIRT6 knockout animals and the genomic instability conferred by reduced levels of SIRT6 in mouse and human cells; several models could conceivably explain this connection [17]. It has been suggested that the organismal effects of SIRT6 deficiency, and similar metabolic phenotypes in other repair-deficient mouse strains, represent a homeostatic response to minimize ongoing genomic damage in the face of a DNA repair defect [34]. Such a model would not be consistent with the sole DNA repair function of mouse SIRT6 being to modulate DNA-PKcs activity, as DNA-PKcs knockout animals do not display a dramatic metabolic phenotype [1]. Reduction of NF-κB function partially rescues the lethality of SIRT6 deficiency [31]; however, survivors still suffer a period of depressed serum glucose, implying that SIRT6 likely plays other roles in metabolism independent of NF-κB. It may be that SIRT6 affects transcription at many loci in a tissue-specific manner; the metabolic defect associated with the lack of SIRT6 could represent the net result of defective regulation of multiple genes. Clearly, much more remains to be learned concerning this fascinating and enigmatic protein.
